# Older Adults’ Expectations of Living in Nursing Home Declined during the Pandemic

**DOI:** 10.1093/geroni/igaf122.3821

**Published:** 2025-12-31

**Authors:** Soohyun Kim, Yulya Truskinovsky, Emily Wiemers

**Affiliations:** Syracuse University, Syracuse, New York, United States; Syracuse University, Syracuse, New York, United States; Syracuse University, Syracuse, New York, United States

## Abstract

The COVID-19 pandemic disproportionately affected nursing homes in the United States. Between March 2020 and January 2022, more than 200,000 nursing home residents died, accounting for roughly one-fourth of all U.S. COVID-19 deaths (Chidambaram, 2022). In addition, residents experienced extreme social isolation and widespread staffing shortages, contributing to physical and psychological decline (Abbassi, 2020), all widely documented in the media. Against this backdrop, we examine how the pandemic shaped community-dwelling older adults’ perceptions of nursing homes. Using data from the Health and Retirement Study (HRS), we analyze how older adults’ self-reported probability of moving to a nursing home within the next five years changed between 2016 and 2022. Our findings show that expectations of future nursing home use declined after the onset of the pandemic. These results are robust across alternative specifications and placebo tests. This study advances understanding of the pandemic’s long-term health implications by highlighting how it shifted expectations about long-term care. Because expectations of nursing home use are predictive of actual utilization, our findings have important implications for long-term care planning. If demand for nursing homes falls faster than anticipated, this may accelerate facility closures while increasing pressure on both unpaid family caregivers and the paid home care workforce.

